# Upcycling of Adlay Bran via *Lactobacillus* Fermentation Enhances Anti-Melanogenic and Antioxidant Activities through MITF/Tyrosinase Pathway Modulation

**DOI:** 10.4014/jmb.2507.07049

**Published:** 2025-11-26

**Authors:** Kyoung Mi Moon, Min-Kyeong Lee, Ji Yun Van, Ah-reum Kim, Su-Yeon Park, Ji Young Hwang, Jaeseong Seo, Jiyun Lee, Jae-Il Kim, Young Mi Lee, Chun Whan Choi, Bonggi Lee

**Affiliations:** 1 Department of Food Science and Nutrition, Pukyong National University, Busan, Republic of Korea; 2 Biotechnology Research Division, National Institute of Fisheries Science, Busan, Republic of Korea; 3 Department of Smart Green Technology Engineering, Pukyong National University, Busan, Republic of Korea; 4 Natural Product Research Team, Biocenter, Gyeonggido Business and Science Accelerator, Gyeonggi-Do, Republic of Korea; 5 Marine Integrated Biomedical Technology Center, The National Key Research Institutes, Pukyong National University, Busan 48513, Republic of Korea

**Keywords:** Adlay bran, melanogenesis, tyrosinase, fermentation

## Abstract

Upcycling agricultural byproducts into high-value functional ingredients represents a sustainable strategy for environmental preservation. Adlay (*Coix lacryma-jobi* var. *ma-yuen*) bran, a nutrient-rich byproduct discarded during seed polishing, was fermented with *Lactiplantibacillus plantarum*, which markedly enhanced its anti-melanogenic activity. The fermented extract suppressed melanin production by modulating the MITF/tyrosinase signaling pathway, with the n-butanol (BuOH) fraction exhibiting the most potent effect. Isoquercitrin and 2-O-β-glucopyranosyl-7-methoxy-2H-1,4-benzoxazin-3(4H)-one were identified as the principal active compounds, and *in silico* analysis revealed strong binding affinities to tyrosinase. Furthermore, fermentation enhanced antioxidant capacity, as evidenced by DPPH and FRAP assays. Collectively, these findings demonstrate that fermented adlay bran constitutes a sustainable bioactive resource with promising applications in skincare and nutraceuticals.

## Introduction

Melanogenesis is a key biological process that protects the skin from ultraviolet (UV) radiation and determines skin pigmentation. This process is regulated by hormones, cytokines, growth factors, and various environmental factors, and maintaining the balance of expression and activity of melanogenic enzymes is crucial. Disruption of this balance can lead to pigmentation disorders such as melasma, freckles, and post-inflammatory hyper-pigmentation [1, 2]. Melanin is synthesized primarily in melanocytes located in the basal layer of the epidermis, where it is produced within specialized organelles called melanosomes. In these organelles, major enzymes such as tyrosinase, tyrosinase-related protein 1 (*TRP-1*), and tyrosinase-related protein 2 (*TRP-2*) catalyze a series of reactions converting the amino acid tyrosine into melanin [1, 2].

Despite the development of numerous skin-lightening products driven by the growing interest in skin aesthetics, there remains a strong demand for novel agents that can regulate pigmentation safely and effectively. In particular, natural products derived from plants, with their diverse chemical compositions and bioactivities, have attracted considerable attention as potential skin-whitening agents. Among them, adlay (*Coix lacryma-jobi* var. *ma-yuen*) bran extracts have been reported to possess promising skin-lightening properties [3].

According to the Food and Agriculture Organization (FAO), agricultural residue burning, as part of open-field biomass combustion, contributes to 13.6% of global PAH emissions from 1960 to 2030 [4]. In response, the United Nations Economic Commission for Europe (UNECE) highlighted the importance of emission control measures and alternative solutions during its 41st session in 2021 [5]. A study has shown that[6], the bran by-product constitutes a significant portion of the adlay grain, yet its utilization remains limited. Given that global adlay production reached over 4.6 million metric tons in 2020, the potential volume of discarded bran is substantial, underscoring the need for innovative valorization strategies. In this study, we aimed not only to ferment adlay bran and examine its bioactivities but also to design this process as a sustainable upcycling strategy. Specifically, we sought to demonstrate, through biological evidence, that low-value agricultural byproducts such as adlay bran can be transformed into skin-beneficial materials via fermentation, thereby reducing environmental burdens and enhancing industrial value.

Adlay is a versatile soft-shelled seed crop widely cultivated across Asia, valued for its nutritional content and traditional uses as food, medicine, and dietary supplement due to its bioactive compounds [7]. Research has highlighted the enhanced biofunctional properties of adlay bran, including anti-haemolysis [8], anti-proliferative [9], and xanthine oxidase-inhibitory activities [10]. Fermentation, a process commonly employed in the production of fermented foods, is known to enhance these properties by improving flavor, preservation, and physiological benefits, particularly antioxidant efficacy [11, 12]. However, studies comparing adlay bran and fermented adlay bran for skin health-related functionalities need to be elucidated. Moreover, since adlay bran is typically discarded during seed polishing, its transformation into bioactive-rich extracts represents a sustainable upcycling approach. Thus, in this study, we not only aimed to evaluate the impact of fermentation on skin health-associated functionalities of adlay bran, but also to highlight this process as a practical strategy for valorizing agricultural byproducts. Fermentation was carried out using *Lactiplantibacillus plantarum*, a versatile and widely studied bacterium commonly found in fermented foods and the human gastrointestinal tract [13].

To delve deeper into the effects of fermentation, we analyzed the solvent fractions of fermented adlay bran extracts using high-performance liquid chromatography (HPLC) to identify bioactive compounds responsible for the observed functionalities. These analyses were complemented by various cell-free and cellular experiments, which provided insights into the mechanisms underlying its skin health-related benefits, such as melanogenesis inhibition and antioxidant activity.

## Materials and Methods

### Materials

The following chemicals were obtained from Sigma-Aldrich (USA): 2,2-diphenyl-1-picrylhydrazyl, 2,4,6-Tris(2-pyridyl)-s-triazine, Iron(II) sulfate heptahydrate, Iron(III) chloride hexahydrate, mushroom tyrosinase, L-tyrosine, L-DOPA, phenylmethylsulfonyl fluoride, α-melanocyte stimulating hormone (α-MSH), and Triton X-100. All items related to cell culture were procured from Welgene (Republic of Korea).

### Preparation of Fermented Adlay Bran Extracts

Adlay bran was sourced from a mill in Yeoncheon, a prominent adlay-growing region in Korea. To prepare the bran for use, it was combined with water at a 20:80 weight ratio, forming a sludge that was then sterilized at 121°C for 20 min. The protocols used for extraction were adapted from existing literature [3].

For microbial fermentation, *L. plantarum* was cultured in 50 ml of De Man, Rogosa, and Sharpe (MRS) broth under anaerobic conditions at 37°C for 16 h. Following this, the sterilized adlay bran sludge was inoculated with 0.1% (v/v) of the *L. plantarum* culture and allowed to ferment statically at 37°C for 48 h. A control batch was prepared by incubating the same adlay bran sludge without bacterial inoculation under identical conditions.

The fermented and non-fermented adlay bran was then subjected to extraction using 70% ethanol in a 1:3 volume ratio for 24 h at ambient temperature. Post-extraction, the solids were removed via centrifugation at 6,000 rpm for 20 min, and the clear supernatant was filtered using Whatman No. 1 filter paper, evaporated, and lyophilized at -80°C for 24 h. The resultant freeze-dried powder from both the fermented (FAB) and non-fermented bran (NFAB) was stored at -20°C for subsequent analysis.

To further isolate specific compounds, the residual extract was partitioned sequentially with methylene chloride (MC), ethyl acetate (EA), and n-butyl alcohol (BuOH). The BuOH fraction was then processed through medium-pressure liquid chromatography (MPLC) and preparative high-performance liquid chromatography (HPLC) to isolate and identify distinct compounds.

### Spectroscopy of Isolated Compounds

The BuOH fraction (5 g) was placed in C18 gel (120 g) column (Biotage sfär C18D) chromatography, eluted with acetonitrile in H_2_O according to a step-gradient manner (1% to 100%) to give six fractions (F1- F6). We purified compound **1** (5.5 mg) from fraction F2 through repeated JAI-GS310 chromatography, employing an isocratic elution with 80% methanol (MeOH). Finally, we purified compound **2** (4.2 mg) from repeated RP-18 chromatography of F5 with step-gradient elution of MeOH in H_2_O. By comparison with NMR spectral data and literature [14, 15], the isolated compounds were identified as 2-O-β-glucopyranosyl-7-methoxy-2H-1,4-benzoxazin-3(4H)-one and isoquercitrin.

**2-O-β-Glucopyranosyl-7-methoxy-2H-1,4-benzoxazin-3(4H)-one.** Brown gum; ^1^H-NMR (700 MHz, D2O) δ 6.85 (1H, d, J = 9.1 Hz, H-5), 6.68 (1H, d, J = 1.4 Hz, H-8), 6.62 (1H, d, J = 8.4 Hz, H-6), 5.70 (1H, s, H-2), 4.74 (1H, d, J = 8.4 Hz, H-1'), 3.78 (1H, d, J = 11.9 Hz, H-6'b), 3.70 (3H, s, -OCH3), 3.59 (1H, dd, J = 5.6, 11.9 Hz, H-6'a), 3.40 (2H, dd, J = 9.1, 18.2 Hz, H-3', H-5'), 3.26 (1H, t, J = 9.8 Hz, H-4'), 3.14 (1H, t, J = 8.4 Hz, H-2'); ^13^C-NMR (175 MHz, D_2_O) δ 161.4 (C-3), 156.2 (C-7), 140.6 (C-9), 118.2 (C-10), 117.0 (C-5), 109.5 (C-6), 103.7 (C-8), 102.0 (C-1'), 95.4 (C-2), 76.3 (C-5'), 75.3 (C-3'), 72.8 (C-2'), 69.1 (C-4'), 60.5 (C-6'), 55.7 (-OCH_3_) ; ESIMS (positive) m/z 356 [M – H]^–^.

**Isoquercitrin.** Yellow amorphous powder; ^1^H-NMR (700 MHz, CD_3_OD) δ 7.71 (1H, d, J = 2.1 Hz, H-2'), 7.58 (1H, dd, J = 8.4, 2.1 Hz, H-6'), 6.86 (1H, d, J = 8.4 Hz, H-5'), 6.38 (1H, d, J = 2.1 Hz, H-8), 6.19 (1H, d, J = 1.4 Hz, H-6), 5.25 (1H, d, J = 7.7 Hz, H-1''), 3.72 (1H, dd, J = 11.9, 2.1 Hz, H-6''a), 3.58 (1H, dd, J = 11.9, 5.6 Hz, H-6''b), 3.49 (1H, m, H-2''), 3.43 (1H, t, J = 9.1 Hz, H-3''), 3.35 (1H, m, H-4''), 3.23 (1H, m, H-5''); ^13^C-NMR (175 MHz, CD3OD) δ 179.5 (C-4), 166.0 (C-7), 163.1 (C-5), 159.1 (C-9), 158.5 (C-2), 149.9 (C-4), 145.9 (C-3'), 135.7 (C-3), 123.2 (C-1'), 123.1 (C-6'), 117.6 (C-5), 116.0 (C-2'), 105.7 (C-10), 104.4 (C-1''), 99.9 (C-6), 94.8 (C-8), 78.4 (C-3''), 78.2 (C-5''), 75.8 (C-2''), 71.3 (C-4''), 62.6 (C-6''); ESI-MS m/z 465 [M + H]^+^.

### Tyrosinase Activity Assay Using Mushroom Tyrosinase

The activity assay of cell-free mushroom tyrosinase was conducted using L-tyrosine as a substrate, referring to a previously published method [16]. Briefly, various concentrations of non-fermented adlay bran extract (ABE) and adlay bran fermented extract (ABFE), methylene chloride (MC), Water fraction, ethyl acetate (EA) fraction, and n-butyl alcohol (BuOH) fraction (10, 50, 100, 200, and 400 ppm) were distributed in a 96-well plate. Subsequently, a 50 mM sodium phosphate buffer (pH 6.5) containing 1 mM L-tyrosine was added to each well. Then, 5 μl of 1,000 U/ml mushroom tyrosinase was added to each well, the plate was incubated for 30 min, and the absorbance was measured at 490 nm using a microplate reader (AMR-100, Allsheng, China).

### DPPH Radical Scavenging Activity Assay

The antioxidant activity of the fermented adlay bran extracts by fraction was determined using a previously established method [17]. Briefly, we investigated its capability to quench free radicals using 2,2-diphenyl-1-picrylhydrazyl (DPPH). Each fraction of the fermented adlay bran extracts was dispensed into individual wells of a 96-well plate at concentrations ranging from 1 to 100 ppm. A 1M DPPH solution was prepared by dissolving DPPH in 80% methanol to achieve a final concentration of 0.1 mM, and this DPPH solution was subsequently added. The plates were then shielded from light and incubated for 30 min at room temperature. Following the incubation period, absorbance at 510 nm was measured using a microplate reader, and optical density values were calculated.

### Ferric Reducing Antioxidant (FRAP) Assay

The reducing power of each fraction from the fermented adlay bran extract was determined using a previously established method [18]. The fermented adlay bran extract was distributed into each well of a 96-well plate for each fraction, with concentrations ranging from 1 to 100 ppm. The FRAP working solution was prepared by mixing 300 mM acetic acid buffer (pH 3.6), 10 mM 2,4,6-Tris(2-pyridyl)-s-triazine, 20 mM ferric chloride, and distilled water in a ratio of 10:1:1:1.2. Subsequently, the plate was incubated at 37°C for 10 min. After the incubation period, absorbance at 510 nm was measured using a microplate reader. Ferrous sulfate was measured at the same concentration as each fraction of adlay bran extract, and a standard curve was constructed using the average FRAP value versus concentration. This was used to calculate the antioxidant capacity of each fraction of adlay bran extract.

### Cell Culture and Viability Assay

The B16F10 mouse melanoma cell line was maintained in a controlled environment at 37°C with 5% CO_2_ in DMEM medium supplemented with 10% fetal bovine serum and 1% penicillin/streptomycin. Cell viability was assessed using the CellTiter96 AQueous One Solution Cell Proliferation Assay Kit (MTS Assay) procured from Promega (USA) according to established protocols [9]. In brief, B16F10 cells were seeded at a density of 1 × 10^4^ cells per well in 96-well plates. After a 24-h incubation, cells were exposed to varying concentrations (6.25, 12.5, 25, 50, 100, and 200 ppm) of MC, EA, BuOH, and water fractions of the fermented adlay bran extracts. Following a 1-h incubation with 5 μl of MTS reagent, absorbance was measured at 490 nm using a microplate reader.

### Melanin Contents in B16F10 Melanoma Cells

The melanin content was determined by referring to previously published studies [18]. Briefly, B16F10 cells were treated with different concentrations of ABE (15, 50, and 100 ppm), ABFE (15, 50, and 100 ppm), MC fraction (1, 5, and 15 ppm), water fraction (15, 50, and 200 ppm), EA fraction (5, 15, and 50 ppm), and BuOH fraction (1, 5, and 15 ppm). Arbutin, used as a positive control, was applied at the highest concentration of each sample group. One hour later, they were additionally treated with 1 μM α-MSH and cultured for 6 days. After the incubation period, cells were washed with PBS and collected by treatment with 1N NaOH solution, followed by lysing for 1 h at 60°C. Subsequently, the lysed cells were transferred to a 96-well plate, and the absorbance was measured at 490 nm. Relative melanin content was determined by normalizing melanin values to the total protein content.

### Cellular Tyrosinase Activity Assay

The intracellular tyrosinase activity assay was performed according to previously established methods [18]. B16F10 cells (5 × 10^3^ cells/well) were exposed to BuOH (5 and 15 ppm) for 1 h, followed by stimulation with 1 μM α-MSH for 6 days. Post-treatment, cells were lysed in a 50 mM sodium phosphate buffer containing 1% Triton X-100 and 0.1 mM phenylmethylsulfonyl fluoride. The cell lysates were then subjected to freezing at -80°C for 30 min, followed by centrifugation at 12,000 ×*g* to obtain the supernatant. Subsequently, the supernatant was mixed with L-DOPA at a concentration of 2 mg/ml and incubated at 37°C for 1 h. Absorbance readings were recorded at 492 nm. Intracellular tyrosinase activity was evaluated based on absorbance values normalized to the total protein content and expressed as a percentage of the control.

### Real-Time PCR

To obtain a total of 1 μg of RNA, B16F10 melanoma cells were subjected to RNA extraction using RiboEXTM reagent (GeneAll, Republic of Korea). cDNA synthesis was conducted using the Primer Script RT Reagent kit (SMART GENE, Republic of Korea) with 1 μg of total RNA. Quantitative real-time PCR was performed using the TOPrealTM SYBR Green qPCR PreMIX (Enzynomics, Republic of Korea) and the QuantStudioTM 1 Real-Time PCR System (Applied Biosystems, USA). The expression levels of target genes, normalized to β-actin, were determined using the -ΔΔCT method.

### *In Silico* Protein–Ligand Docking Simulation

Docking studies were carried out utilizing AutoDock Vina 1.1.2 (available at http://vina.scripps.edu, accessed on 20 May 2024), focusing on the binding interactions with tyrosinase sourced from Agaricus bisporus (PDB ID: 2Y9X). The compounds tested, arbutin and kojic acid, are recognized for their inhibitory effects on tyrosinase. The computational models simulated how arbutin (CID: 440936) and kojic acid (CID: 3840) interact with tyrosinase at the tropolone binding site. Predictions were also made regarding the potential of isoquercitrin (CID: 5280804) and 2-O-β-glucopyranosyl-7-methoxy-2H-1,4-benzoxazin-3(4H)-one to engage at this site.

To ensure accuracy in molecular representations, structural data for arbutin, kojic acid, and isoquercitrin were retrieved from PubChem, while the 3D configuration for 2-O-β-glucopyranosyl-7-methoxy-2H-1,4-benzoxazin-3(4H)-one was constructed using MolView (https://molview.org, accessed on 20 May 2024). Analysis of the potential molecular interactions between these compounds and tyrosinase was further refined using LigandScout 4.4 software, providing a detailed assessment of the binding dynamics and affinities involved.

### Statistical Analysis

The data are expressed as mean ± standard error of the mean (SEM) from a minimum of three independent experiments. Group differences were assessed utilizing one-way analysis of variance (ANOVA) coupled with Tukey's post-hoc test, executed with GraphPad Prism 5.0 (GraphPad software, USA).

## Results

### Melanogenesis Inhibitory and Antioxidant Activities of Adlay Bran Extract and Fermented Adlay Bran Extract

**Fig. 1:**
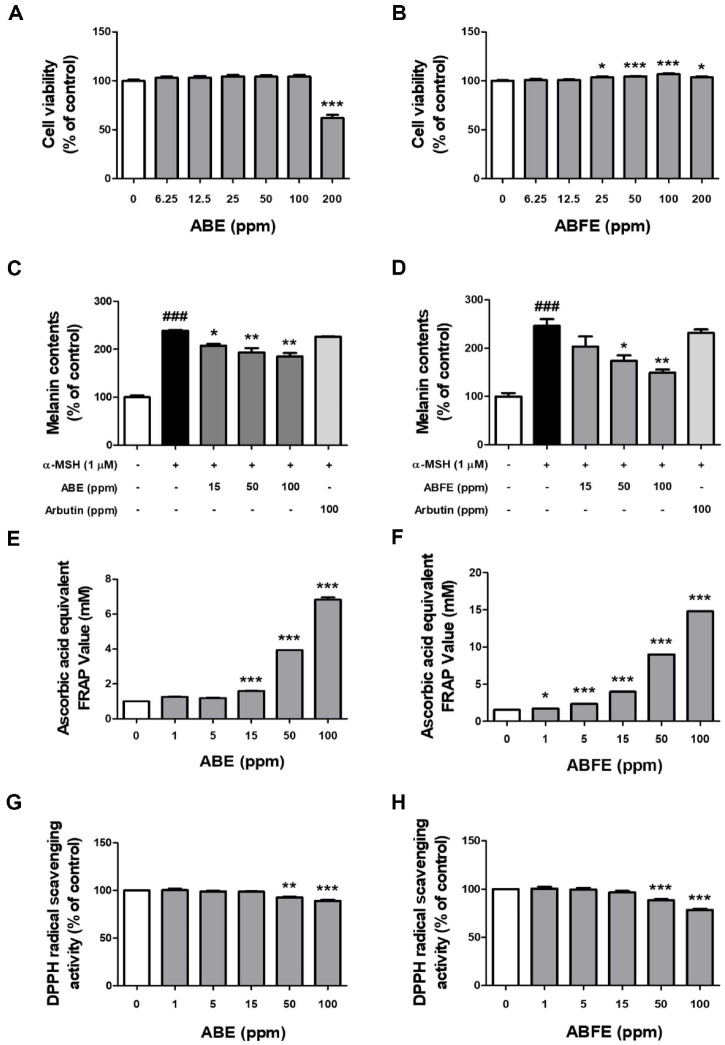
Antioxidant capacity and melanogenesis inhibitory activities of non-fermented adlay bran extract (ABE) and adlay bran fermented extract (ABFE). Cytotoxicity was assessed in B16F10 cells by exposing the cells to increasing concentrations of (**A**) ABE and (**B**) ABFE (6.25, 12.5, 25, 50, 100, and 200 ppm) for 24 h. Intracellular melanin content was quantified in B16F10 cells stimulated with α-MSH after pretreatment with different concentrations of (**C**) ABE and (**D**) ABFE (15–300 ppm), and the results were compared with those obtained using the positive control arbutin (100 ppm) for 1 h. The antioxidant capacity of ABE and ABFE was further evaluated in a cell-free system through the FRAP assay for (**E**) ABE and (**F**) ABFE and the DPPH assay for (**G**) ABE and (**H**) ABFE, with five independent measurements per group (*n* = 5/group). Results are expressed as mean ± SEM, where ^###^*p* < 0.001 indicates a significant difference compared to the untreated control group, and *p < 0.05 and **p < 0.01 indicate a significant difference compared to the α-MSH–treated group.

We investigated the potential inhibitory effects of ABE and ABFE on melanogenesis in B16F10 melanoma cells by assessing melanin content following treatment with α-MSH, a well-known melanogenesis stimulator. Prior to the melanin assay, we conducted an *in vitro* MTS assay to evaluate the cytotoxicity of ABE and ABFE. The results showed no cytotoxic effects in B16F10 melanoma cells at concentrations of up to 100 ppm for both ABE and ABFE (Fig. 1A and 1B). However, at 200 ppm, ABE exhibited cytotoxicity, whereas ABFE did not. Exposure to α-MSH significantly elevated melanin levels in B16F10 melanoma cells. While both extracts exhibited a significant anti-melanogenic effect in a concentration-dependent manner, there were differences observed between the groups. Treatment with ABE reduced melanin levels by 19% at 50 ppm and 23% at 100 ppm, whereas fermented ABE suppressed melanin levels by 29% at 50 ppm and 40% at 100 ppm (Fig. 1C and 1D). These findings suggest that fermentation of adlay bran enhances its anti-melanogenic capacity.

Antioxidants like glutathione and ascorbic acid are known to interrupt the chain reaction and impede melanin formation by reacting with intermediate products [19, 20]. Therefore, we evaluated the antioxidant activities of ABE and ABFE using the ferric reducing antioxidant power assay and DPPH radical scavenging assay, which are commonly used methods to assess the antioxidant capacity of natural products. While both ABE and ABFE demonstrated iron-reducing power and DPPH radical scavenging activity, the fermented extract exhibited a more pronounced effect (Fig. 1E and 1H). These data indicate that the fermentation process enhances the antioxidant capacity of adlay bran.

### Antioxidant Activities of Fractions Obtained from Fermented Adlay Bran Extract

**Fig. 2:**
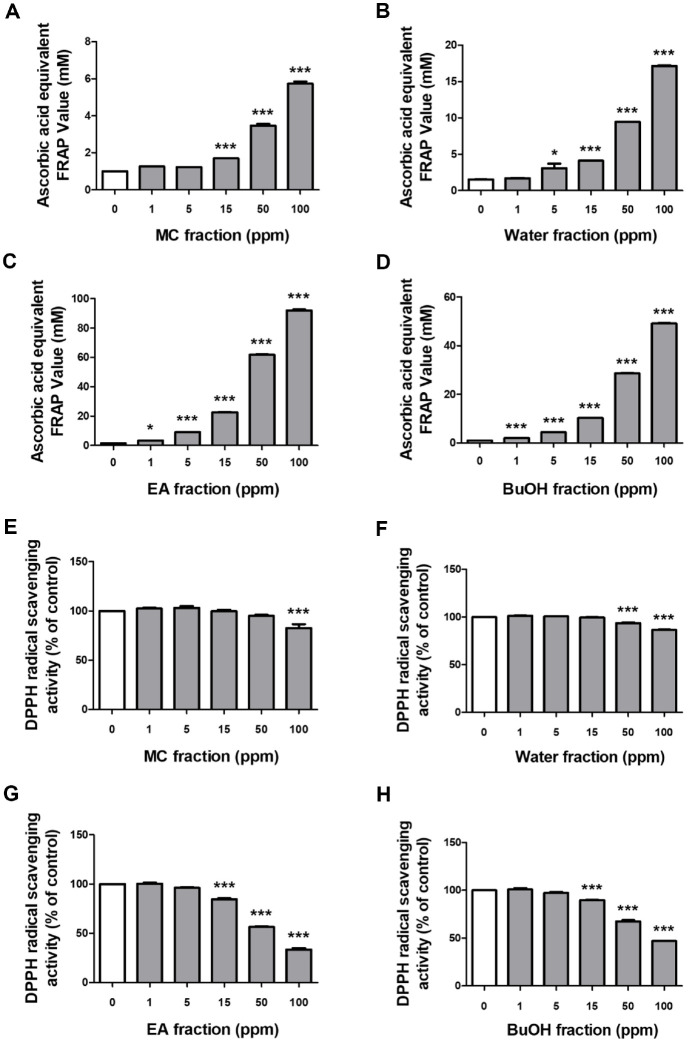
Antioxidant capacity of different fractions derived from fermented adlay bran extracts. The antioxidant activities of fractionated extracts obtained from adlay bran fermentation were determined in a cell-free system with three independent measurements per group (*n* = 3/group). FRAP analysis was conducted for the (**A**) methylene chloride (MC), (**B**) water, (**C**) ethyl acetate (EA), and (**D**) n-butyl alcohol (BuOH) fractions at concentrations ranging from 0 to 100 mM. Free radical scavenging activity was further evaluated using the DPPH assay for the (**E**) MC, (**F**) water, (**G**) EA, and (**H**) BuOH fractions at concentrations ranging from 0 to 100 ppm. Data are presented as mean ± SEM, and statistical significance is indicated as *p < 0.05, **p < 0.01, and ***p < 0.001 compared to the untreated control group.

To identify and isolate the specific components responsible for the observed biological activities, MC, EA, BuOH, and water fractions were fractionated from ABFE and were then analyzed for melanogenesis inhibition and antioxidant activity. The MC, water, EA, and BuOH fractions of the fermented adlay bran extract exhibited excellent iron-reducing activity, among which the EA and BuOH fractions showed the most powerful activity (Fig. 2A-2D). Similar to the iron-reducing power analysis results, the DPPH radical scavenging analysis also confirmed that the EA and BuOH fractions, among the four fractions, showed the most powerful activity (Fig. 2E-2H).

### Anti-Melanogenic Activities of Fractions Obtained from Fermented Adlay Bran Extract

**Fig. 3:**
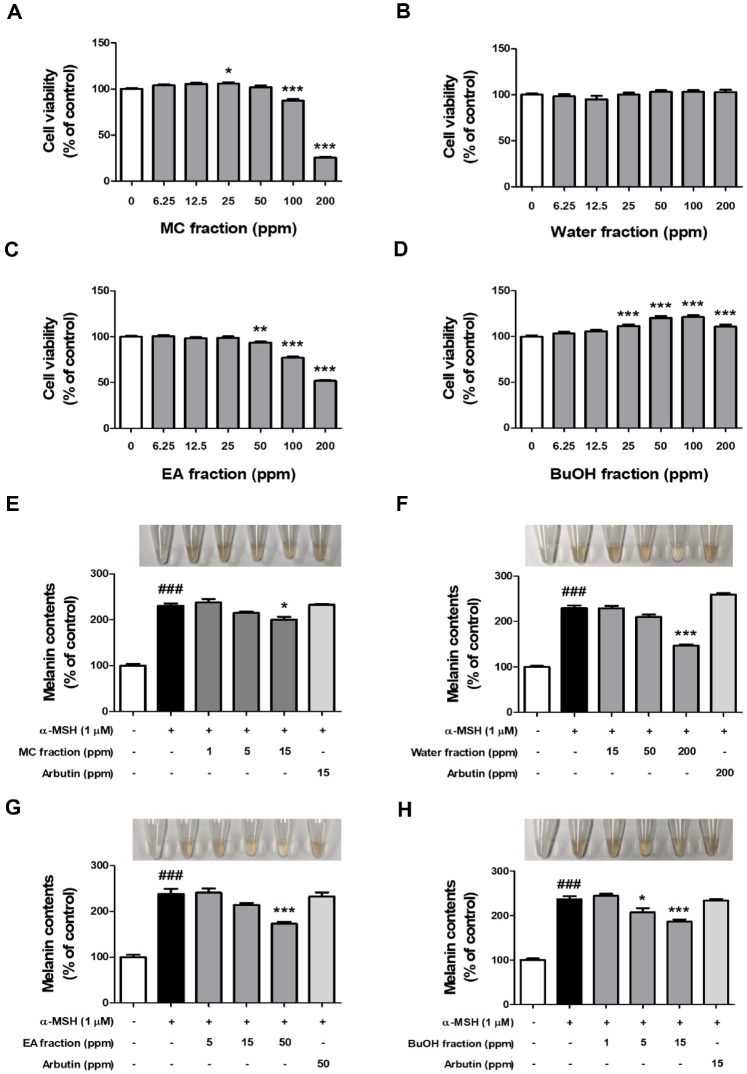
Melanogenesis inhibitory activities of different fractions derived from fermented adlay bran extracts. Cellular toxicity in B16F10 cells was evaluated after 24 h treatment with increasing concentrations of the (**A**) methylene chloride (MC), (**B**) water, (**C**) ethyl acetate (EA), and (**D**) n-butyl alcohol (BuOH) fractions (6.25, 12.5, 25, 50, 100, and 200 ppm), with six independent measurements per group (*n* = 6/group). Statistical significance is indicated as *p < 0.05, **p < 0.01, and ***p < 0.001 compared to the untreated control group. The fraction-specific effects of fermented extracts on melanin production were further examined in α-MSH–stimulated B16F10 cells after pretreatment with (**E**) MC (1, 5, 15 ppm), (**F**) water (15, 50, 200 ppm), (**G**) EA (5, 15, 50 ppm), (**H**) BuOH (1, 5, 15 ppm), and the positive control arbutin (15, 50, 200 ppm; selected to match the maximum concentrations of each fraction) for 1 h, with five independent measurements per group (*n* = 5/group). Results are expressed as mean ± SEM, with ^###^*p* < 0.001 denoting a significant difference compared to the untreated control group, and *p < 0.05, **p < 0.01, ***p < 0.001 indicating significance compared to the α-MSH–treated group.

**Fig. 4:**
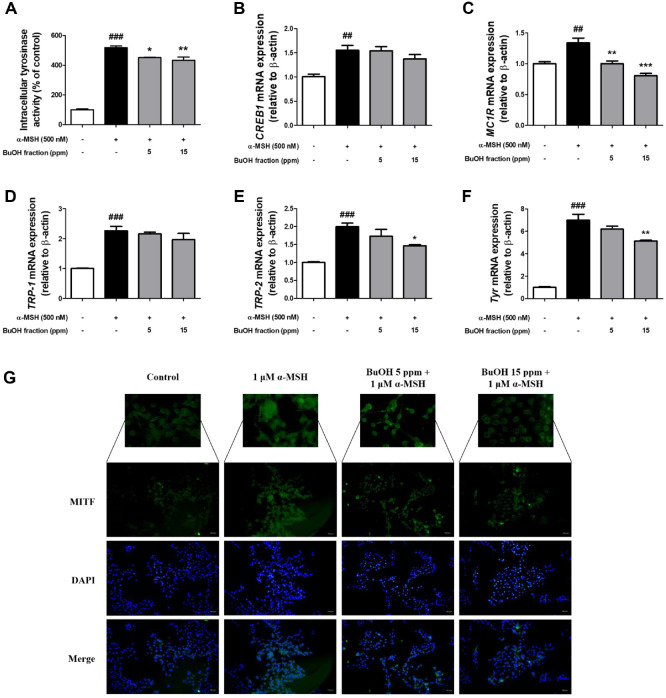
Inhibitory effects of the n-butyl alcohol (BuOH) fraction of fermented adlay bran extract on tyrosinase activity and melanogenesis-related gene expression in B16F10 melanoma cells. (**A**) B16F10 cells were treated with BuOH fractions (5 and 15 ppm) for 1 h, stimulated with α-MSH for 6 days, and then cellular tyrosinase activity was measured. Quantitative PCR was performed to assess the effects of the BuOH fraction on the mRNA expression levels of (**B**) *CREB1*, (**C**) *MC1R*, (**D**) *TRP-1*, (**E**) *TRP-2*, and (**F**) *tyrosinase*. (**G**) *MITF* (green) was visualized by immunofluorescence staining using an FSD™-conjugated secondary antibody, and nuclei (blue) were counterstained with 4',6'-diamidino-2-phenylindole dihydrochloride (DAPI). All data are expressed as mean ± SEM from at least three independent experiments. Statistical significance is indicated as ##*p* < 0.01 and ###*p* < 0.001 compared to the untreated control group, and *p < 0.05, **p < 0.01, and ***p < 0.001 compared to the α-MSH–treated group.

We additionally assessed the potential anti-melanogenic activity of fractions derived from ABFE at non-cytotoxic concentrations (Fig. 3A-3D). In B16F10 melanoma cells stimulated with α-MSH, intracellular melanin production levels significantly increased compared to the control group. However, this increase was significantly suppressed in all four fractions, and in particular, the BuOH fraction was found to most effectively inhibit melanin production induced by α-MSH even at low concentrations (Fig. 3E-3H). Although the EA fraction exhibited slightly stronger antioxidant activity in some assays (Fig. 2C and 2D), the BuOH fraction consistently showed superior inhibitory effects on melanin production in B16F10 cells (Fig. 3E-3H) and on tyrosinase activity (Figs. 4A and S1). While the water fraction displayed some inhibitory activity, its effects were only significant at higher concentrations (200 ppm) and were not consistent at lower concentrations (*e.g.*, 50 ppm). In contrast, the BuOH fraction exerted strong inhibitory effects even at low concentrations (5 and 15 ppm). Based on this comprehensive evaluation, the BuOH fraction was selected for further mechanistic investigation.

### Molecular Mechanisms of the Anti-Melanogenic Effects of BuOH Fraction Obtained from Fermented Adlay Bran Extract

In our cell-free studies, we observed that the BuOH fraction decreases the activity of mushroom tyrosinase (Fig. S1), and from this, we hypothesized that the BuOH fraction may reduce intracellular tyrosinase activity, ultimately inhibiting melanin production. Analysis of intracellular tyrosinase activity in B16F10 cells treated with α-MSH alone or together with the BuOH fraction showed significantly lower tyrosinase activity in cells treated with the BuOH fraction compared to cells treated with α-MSH alone (Fig. 4A). These findings imply that by lowering cellular tyrosinase activity, the BuOH fraction contributes to the inhibition of melanogenesis.

MITF enhances the transcription of key genes like tyrosinase, pivotal for melanin synthesis, driving the process of pigment production [21, 22]. Therefore, to elucidate the molecular mechanisms underlying the anti-melanogenic effects mediated by the BuOH fraction, we measured the mRNA expression levels of *CREB1*, *MC1R*, *TRP-1*, *TRP-2*, and *Tyrosinase*, all of which are involved in melanogenesis (Fig. 4B-4F). α-MSH treatment significantly increased the mRNA levels of *CREB1*, *MC1R*, *TRP-1*, *TRP-2*, and *tyrosinase*, while BuOH fraction treatment significantly decreased the mRNA levels of *MC1R*, *TRP-2*, and *tyrosinase* among these genes (Fig. 4B-4F). Furthermore, immunofluorescence analysis was carried out to assess the suppressive impact of BuOH fraction on the nuclear translocation of MITF, a crucial transcription factor involved in controlling tyrosinase activity (Fig. 4G). Fluorescence microscopy observations indicated that BuOH fraction treatment significantly inhibited the translocation of MITF into the nucleus compared to B16F10 melanoma cells treated exclusively with α-MSH (Fig. 4G). These findings indicate that the BuOH fraction inhibits melanin production by not only inhibiting tyrosinase activity but also by preventing the translocation of MITF into the nucleus, which in turn decreases the mRNA levels of *TRP-2* and *tyrosinase*.

### Single Compound Isolation from the BuOH Fraction

**Fig. 5:**
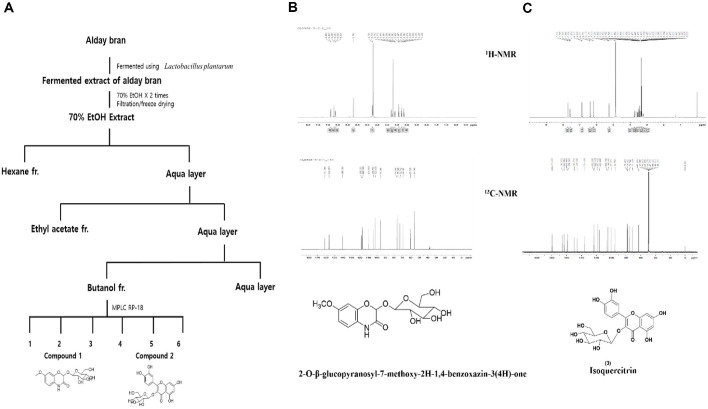
Isolation of single compounds from the n-butyl alcohol (BuOH) fraction of fermented adlay bran extract. (**A**) The procedure for fermenting adlay bran, fractionating the extract, and isolating single compounds. (**B**) Compound 1 (5.5 mg) was isolated from Fraction 2 (F2) through repeated purification using JAI-GS310 chromatography with 80% methanol as the mobile phase. (**C**) Compound 2 (4.2 mg) was purified from Fraction 5 (F5) by RP-18 chromatography with gradient elution of methanol and water. Structural identification of both compounds was performed based on NMR spectroscopic data and comparison with published literature, and the compounds were characterized as 2-O-β-glucopyranosyl-7-methoxy-2H-1,4-benzoxazin-3(4H)-one and isoquercitrin.

The BuOH fraction (5 g) was separated using a C18 gel (120 g) column, yielding six fractions (F1-F6) with acetonitrile in H_2_O elution (1% to 100%). Compound 1 (5.5 mg) was isolated from F2 through repeated JAI-GS310 chromatography with 80% MeOH. Compound 2 (4.2 mg) was purified from F5 using RP-18 chromatography with MeOH in H_2_O elution. Both compounds were identified as DIMBOA-Glc and Isoquercitrin based on NMR data and literature comparison (Fig. 5B-5C). The NMR spectra (^1^H and ^13^C; Fig. 5) confirmed the structural identities, showing characteristic signals for the benzoxazinone backbone in Compound 1 and the flavonoid glycoside framework in compound 2.

### Isoquercitrin and 2-O-β-Glucopyranosyl-7-Methoxy-2H-1,4-Benzoxazin-3(4H)-One May Bind to and Inactivate Tyrosinase

**Fig. 6:**
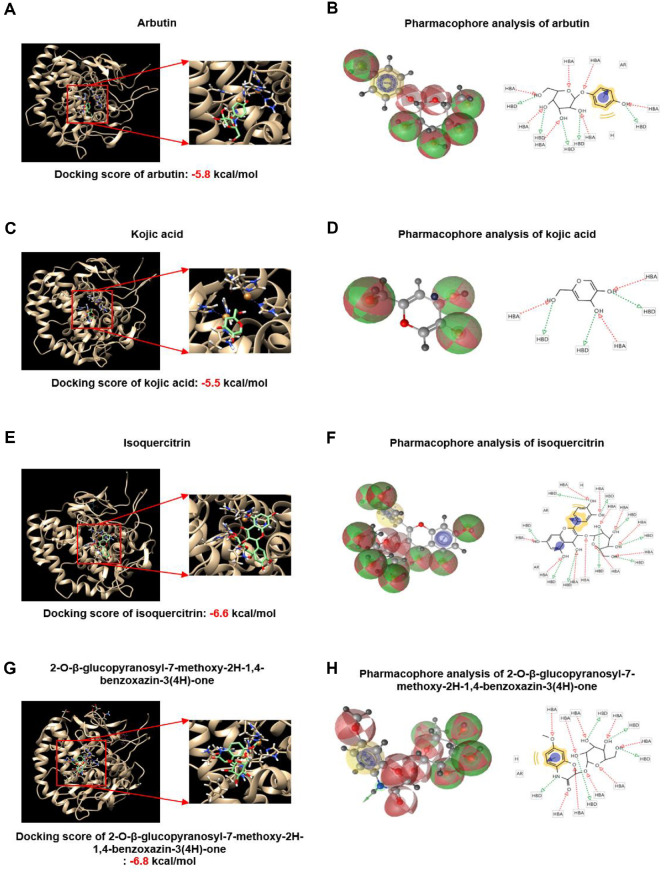
2-O-β-glucopyranosyl-7-methoxy-2H-1,4-benzoxazin-3(4H)-one is a competitive inhibitor of tyrosinase. Binding affinity between tyrosinase (PDB ID: 2Y9X) and (**A**) arbutin (CID: 440936), (**C**) kojic acid (CID: 3840), (**E**) isoquercitrin (CID:5280804), or (**G**) 2-O-β-glucopyranosyl-7-methoxy-2H-1,4-benzoxazin-3(4H)-one. AutoDock Vina software was used to predict the ligand binding capacity of isoquercitrin and 2-O-β-glucopyranosyl-7-methoxy-2H-1,4- benzoxazin-3(4H)-one, which exhibit strong inhibitory activity against tyrosinase. In a marked square box, it represents the tyrosinase binding site with an enlarged image. The binding residue analysis of (**B**) arbutin (CID: 440936), (**D**) kojic acid (CID: 3840), (f) isoquercitrin (CID:5280804), or (**H**) 2-O-β-glucopyranosyl-7-methoxy-2H-1,4-benzoxazin-3(4H)-one with tyrosinase was performed by pharmacophore analysis.

The molecular docking scores for arbutin, kojic acid, isoquercitrin, and 2-O-β-glucopyranosyl-7-methoxy-2H-1,4-benzoxazin-3(4H)-one were analyzed to evaluate their binding affinities to tyrosinase. The results revealed docking scores of -5.8, -5.5, -6.6 kcal/mol, and -6.8 kcal/mol, respectively, indicating their potential as tyrosinase inhibitors (Fig. 6A, 6C, 6E, and 6G). Additionally, both isoquercitrin and 2-O-β-glucopyranosyl-7-methoxy-2H-1,4-benzoxazin-3(4H)-one showed docking profiles suggestive of inhibitory potential towards tyrosinase, which are supportive but not conclusive findings. Consistent with these predictions, both cell-free tyrosinase activity assays (Fig. S1) and intracellular tyrosinase assays in B16F10 cells (Fig. 3A) confirmed that the BuOH fraction significantly inhibited tyrosinase activity.

To elucidate the specific interactions facilitating these binding affinities, binding mode analyses were conducted. Using LigandScout 4.4.8, it was determined that arbutin is likely to engage in binding through seven hydrogen bond acceptors and five hydrogen bond donors, supplemented by one aromatic and one hydrophobic interaction with tyrosinase (Fig. 6B). Kojic acid, on the other hand, appears to form interactions through three hydrogen bond acceptors and donors each (Fig. 6D). Isoquercitrin exhibits more complex binding with eleven hydrogen bond acceptors and nine donors, alongside two aromatic and one hydrophobic interactions (Fig. 6F). Finally, 2-O-β-glucopyranosyl-7-methoxy-2H-1,4-benzoxazin-3(4H)-one engages with tyrosinase through nine hydrogen bond acceptors and five donors, in addition to one hydrophobic and one aromatic interaction (Fig. 6H). Compared with the relatively simple interaction patterns of arbutin and kojic acid, the extensive hydrogen bonding and additional aromatic/hydrophobic contacts observed for isoquercitrin and the benzoxazinone derivative suggest a more stable and specific binding within the tyrosinase active site. This higher degree of interaction is consistent with their lower docking scores and may explain their stronger inhibitory potential relative to conventional inhibitors. These detailed insights into the molecular interactions provide supportive implications for the potential design of tyrosinase inhibitors.

## Discussion

Although adlay seeds are an edible crop and are also used as traditional medicine and dietary supplement due to various functional compounds within [7], adlay bran is one of the wastes produced during adlay refining processes [6]. To increase the value of this agricultural residue, we compared the biological activities of ABE and ABFE. Our data revealed that fermentation with *L. plantarum* markedly elevated the total phenol content and antioxidant capacity of adlay bran ethanol extract. *L. plantarum*–mediated fermentation has also been shown to enhance the functionality of other natural products, exemplified by increased phenolic and flavonoid contents, antioxidant activity, and enzyme inhibition in Jamaican cherry juice and Graptopetalum paraguayense [23, 24]. Moreover, since *L. plantarum* is generally recognized as safe [24], its use represents a practical strategy to strengthen the bioactivity of foods and natural products. Our data showed that ABFE using the *L. plantarum* had better melanin production inhibition and antioxidant activity than ABE (Fig. 1C-1H). These results are consistent with previous reports showing that fermented extracts exhibit greater health benefits than non-fermented ones, which can be attributed to enhanced bioavailability of nutrients, accumulation of bioactive compounds, and the generation of new metabolites during the fermentation process [24, 25].

Since oxidative stress is a critical driver of melanogenesis, mainly by upregulating MITF and melanogenic enzymes such as tyrosinase and TRP1/2 [26, 27], the reinforced antioxidant capacity of ABFE may indirectly contribute to its antimelanogenic effects. For comparison, we tested arbutin, a widely used skin-whitening agent, at the same concentration used for ABFE treatment. Arbutin has been reported to inhibit tyrosinase with an IC_50_ of ~0.5–1.0 mM in cellular assays [28, 29]. In our study, however, arbutin at this concentration showed little effect, whereas ABFE exhibited significant antimelanogenic activity under identical conditions. This contrast suggests that arbutin may require higher concentrations for efficacy, while highlighting the superior activity of ABFE.

In our study, two major compounds, isoquercitrin and DIMBOA-Glc, were identified from the butanol fraction. Isoquercitrin has been repeatedly reported as a key constituent of extracts with anti-melanogenic activity [30-35]. For example, Morus nigra leaf extract standardized to isoquercitrin exhibited potent tyrosinase inhibition, and fermented Polygonum tinctorium extract reduced melanin synthesis by downregulating MITF and melanogenic enzymes via ERK/AKT signaling [30]. Isoquercitrin has also been cited to suppress tyrosinase expression at the cellular level with an IC_50_ of ~21.7 μM [35], suggesting that it likely contributed to the inhibitory effects observed in our fraction. Nevertheless, direct validation with purified isoquercitrin remains limited and warrants further investigation. By contrast, DIMBOA-Glc is a plant-derived benzoxazinone compound that has not previously been linked to melanogenesis. However, structurally related molecules such as DIBOA and MBOA (coixol) have been shown to inhibit tyrosinase through copper chelation and direct enzymatic suppression [32, 33], while synthetic 2-MBO derivatives also display strong anti-tyrosinase and anti-melanogenic activity [35]. Based on these analogs, the detection of DIMBOA-Glc in our active fraction may represent a novel finding with potential anti-melanogenic relevance, which should be further confirmed through studies using purified compounds. Furthermore, molecular docking suggested that both isoquercitrin and DIMBOA-Glc may stably interact with tyrosinase, consistent with their observed inhibitory effects. Although these *in silico* results provide supportive evidence, they remain predictive and require future biochemical validation.

This research aimed to address the broader goal of increasing the practical utilization of adlay bran, an agricultural byproduct, by exploring its potential as a sustainable resource. By repurposing this often-discarded material into a value-added product with applications in skincare and health, our study not only highlights the functional benefits of fermented adlay bran but also contributes to the sustainable management of agricultural residues.

In conclusion, this study demonstrates that fermentation of adlay bran with *L. plantarum* significantly enhances its functionality, particularly in melanogenesis modulation and antioxidant activity. Fermented adlay bran extract exhibited superior efficacy compared to the non-fermented extract, with the BuOH fraction showing the strongest activities. Two bioactive compounds identified from this fraction, isoquercitrin and 2-O-β-glucopyranosyl-7-methoxy-2H-1,4-benzoxazin-3(4H)-one, displayed strong binding affinity to tyrosinase, suggesting their contribution to the observed effects. Collectively, these findings underscore the potential of fermented adlay bran extract as a natural ingredient with promising applications in skincare and nutraceuticals, while also highlighting its role as a sustainable upcycling strategy that adds value to agricultural byproducts.

## Supplemental Materials

Supplementary data for this paper are available on-line only at http://jmb.or.kr.
