# Supplementary material for: Upcycling of Adlay Bran via Lactobacillus Fermentation Enhances Anti-Melanogenic and Antioxidant Activities through MITF/Tyrosinase Pathway Modulation
Source: J Microbiol Biotechnol. 2025 Nov 26;35:e2507049. doi: 10.4014/jmb.2507.07049 (PMC12685594; doi:10.4014/jmb.2507.07049)
Supplement: Supplementary file 1 [file jmb-35-e2507049-supple.pdf]

J. Microbiol. Biotechnol. 2025. 35: e2507049

https://doi.org/10.4014/jmb.2507.07049

Supplementary Figure

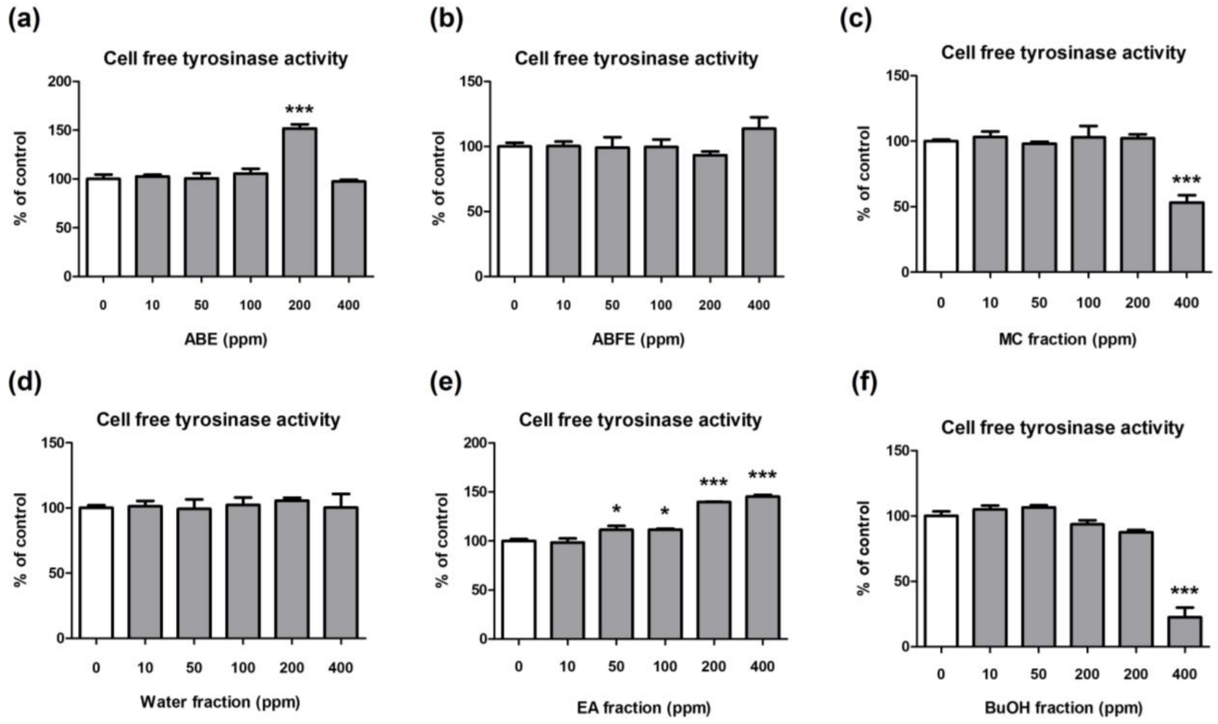

Fig. S1. Cell free tyrosinase activity The inhibitory effect of (a) ABE (b) ABFE (c) MC fraction (d) water fraction (e) EA fraction and (f) BuOH fraction on mushroom tyrosinase activity was measured, and L-tyrosine was used as the substrate. All data represented the mean ± SEM at least three times. $^{*}p < 0.05$, $^{***}p < 0.001$ compared with the non-treated control group.
